# Molecular detection of *Rickettsia* in fleas from micromammals in Chile

**DOI:** 10.1186/s13071-020-04388-5

**Published:** 2020-10-17

**Authors:** Lucila Moreno-Salas, Mario Espinoza-Carniglia, Nicol Lizama-Schmeisser, Luis Gonzalo Torres-Fuentes, María Carolina Silva-de La Fuente, Marcela Lareschi, Daniel González-Acuña

**Affiliations:** 1 Universidad de Concepción, Facultad de Ciencias Naturales y Oceanográficas, Concepción, Chile; 2 Centro de Estudios Parasitológicos y de Vectores CEPAVE (CONICET CCT-La Plata-UNLP), La Plata, Argentina; 3 Universidad Austral de Chile, Facultad de Ciencias, Valdivia, Chile; 4 Universidad de Concepción, Facultad de Ciencias Veterinarias, Chillán, Chile; 5 Facultad de Ciencias Veterinarias, Universidad Austral de Chile, Valdivia, Chile

**Keywords:** Pathogen, Bacteria, Vectors, Fleas, Rodents, Marsupials

## Abstract

**Background:**

Rickettsial diseases are considered important in public health due to their dispersal capacity determined by the particular characteristics of their reservoirs and/or vectors. Among the latter, fleas play an important role, since the vast majority of species parasitize wild and invasive rodents, so their detection is relevant to be able to monitor potential emerging diseases. The aim of this study was to detect, characterize, and compare *Rickettsia* spp. from the fleas of micromammals in areas with different human population densities in Chile.

**Methods:**

The presence of *Rickettsia* spp. was evaluated by standard polymerase chain reaction (PCR) and sequencing in 1315 fleas collected from 1512 micromammals in 29 locations, with different human population densities in Chile. A generalized linear model (GLM) was used to identify the variables that may explain *Rickettsia* prevalence in fleas.

**Results:**

DNA of *Rickettsia* spp. was identified in 13.2% (174 of 1315) of fleas tested. Fifteen flea species were found to be *Rickettsia*-positive. The prevalence of *Rickettsia* spp. was higher in winter, semi-arid region and natural areas, and the infection levels in fleas varied between species of flea. The prevalence of *Rickettsia* among flea species ranged between 0–35.1%. Areas of lower human density showed the highest prevalence of *Rickettsia*. The phylogenetic tree showed two well-differentiated clades with *Rickettsia bellii* positioned as basal in one clade. The second clade was subdivided into two subclades of species related to *Rickettsia* of the spotted fever group.

**Conclusions:**

To our knowledge, this is the first report of the occurrence and molecular characterization of *Rickettsia* spp. in 15 flea species of micromammals in Chile. In this study, fleas were detected carrying *Rickettsia* DNA with zoonotic potential, mainly in villages and natural areas of Chile. Considering that there are differences in the prevalence of *Rickettsia* in fleas associated with different factors, more investigations are needed to further understand the ecology of *Rickettsia* in fleas and their implications for human health.
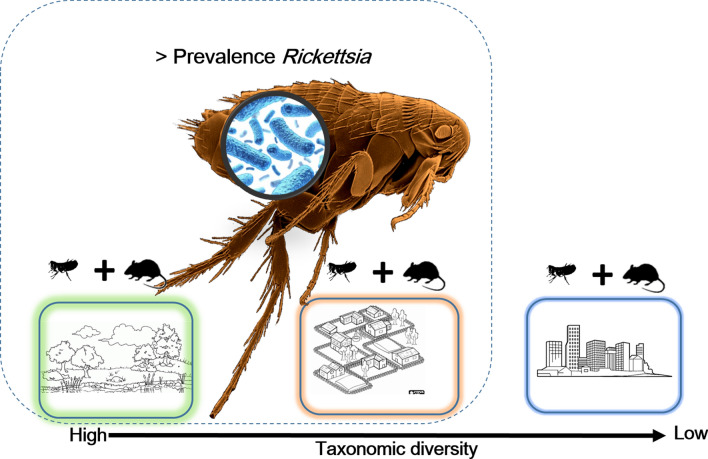

## Background

*Rickettsia* spp. are obligate intracellular microorganisms, Gram-negative coccobacilli, with the ability to reproduce, both in the nucleus and in the cytoplasm of infected cells [1]. These bacteria have a vertebrate reservoir and an arthropod vector (e.g. ticks, mites, fleas and lice); in some cases, the latter may be affected by these bacteria [2]. They have a worldwide distribution and are the causative agents of serious human infections [3].

Currently, 32 species are recognized (http://www.bacterio.net/-allnamesmr.html), and there are many strains that have not yet been characterized, while subspecies and uncultivated species are classified as “*Candidatus*” [4]. Recently, using new classification methods based on formal order analysis (FOA), which considers whole-genome sequencing analysis, two groups are recognized within the genus *Rickettsia*: the major typhus group (MTG) and major spotted fever group (MSFG). The MTG is divided into the typhus group (TG) and ancestral group (AG) and is transmitted by insects. MSFG includes the *R. felis* group, *R. akari* group, and the “classical” spotted fever group that includes several species transmitted by mites and hard ticks, of which the most important are *R. rickettsii* and *R. conorii*, that cause Rocky Mountain spotted fever and Mediterranean spotted fever, respectively [4]. Since *Rickettsia* research has focused on species that affect humans, other species have received less attention [5]. Thus, there are several species of rickettsiae identified and are exclusively associated with arthropods. They are without known secondary hosts and associated with other organisms such as herbivorous insects, leeches, amoebas, inclusive algae, and plants, indicating that these are more common than suspected [5, 6], and that the effects they could cause in humans when contact is made are unknown.

Worldwide, micromammals, and especially rodents, are the main flea hosts. It is recognized that 74% of known flea species parasitize them; therefore, rodents play a fundamental role in the spread of flea-borne diseases, as various species of rodent fleas can also parasitize humans [7]. In addition to this, many rodent species are capable of inhabiting wild environments and adapting to rural and urban environments, which could favor a continuous gradient of transmission between domestic and wild species, and humans [8, 9]. In Chile, despite the great diversity of described fleas (114 species), which mainly parasitize rodents [10, 11], a scarce number of studies have detected *Rickettsia* in fleas [12–15]. These studies have focused on the molecular detection of pathogens in fleas of domestic mammals, identifying *R. felis* from cat and dog fleas (*Ctenocephalides felis* and *C. canis*) in central (Metropolitan region) and southern Chile (Valdivia) [12–14]. Recently, “*Candidatus* Rickettsia asembonensis”, “*Candidatus* Rickettsia senegalensis”, and *R. felis*, were detected in *C. felis* from cats in the Easter Island (Rapa Nui) [15]. No studies have shown their presence in rodent fleas. If this adds to the expansion of the human population invading wild areas, the chance of contacting fleas on infected rodents increases. Since, in some places, peri-urban rodents provide a link between wild rodent and human communities, humans are exposed to some zoonotic agents that circulate in these natural ecosystems [16, 17].

The aim of this study was to detect, characterize, and compare *Rickettsia* spp. from the fleas of micromammals in areas with different human population densities in Chile. The findings will provide the baseline for the future surveillance of *Rickettsia* spp. in Chile.

## Methods

### Sample localities and micromammal-trapping procedures

**Fig. 1:**
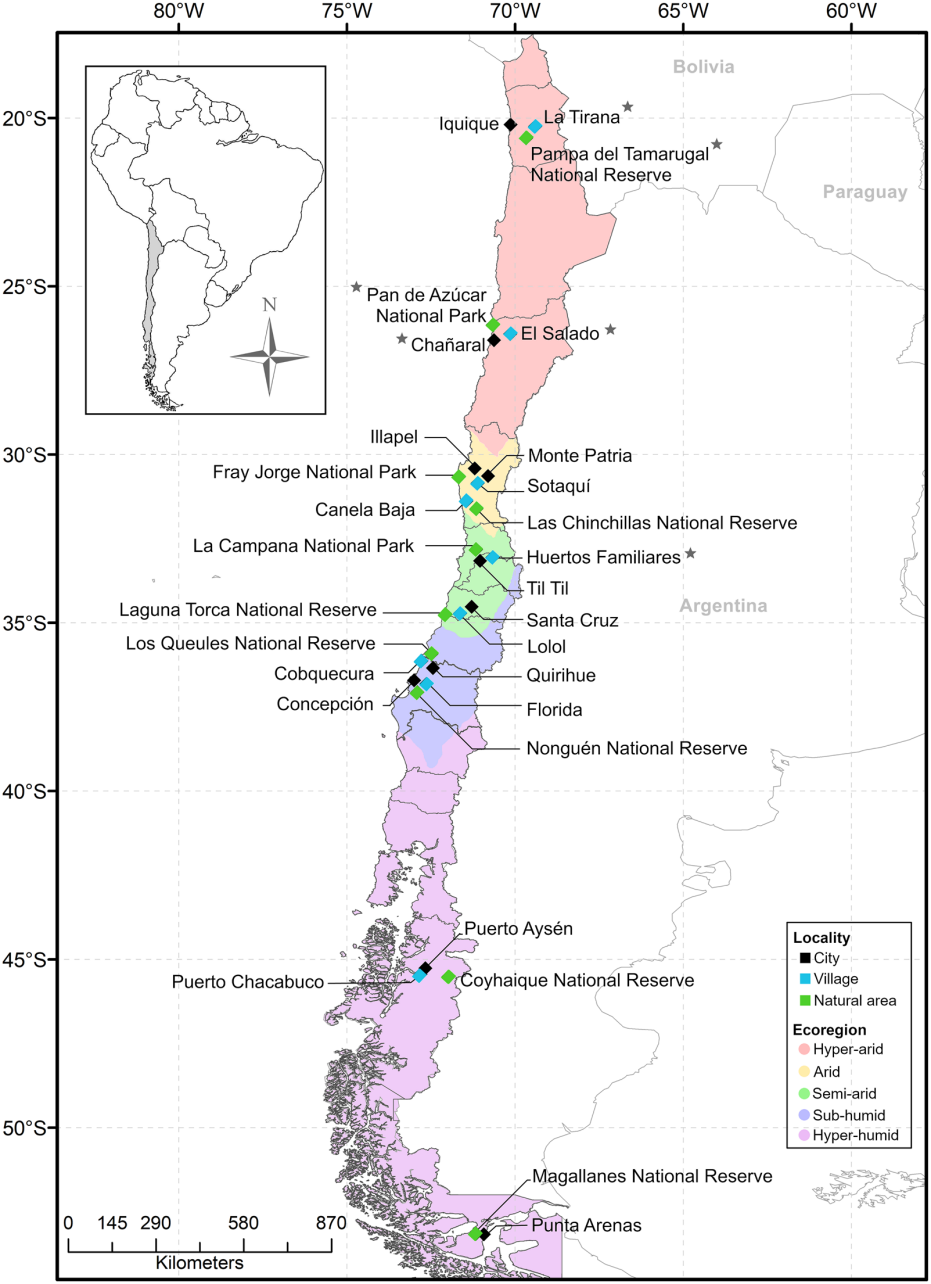
Study area. There are indicated the type of locality where the micromammals were collected. The stars indicate the locations where rodents were not captured

A total of 1512 micromammals belonging to 18 species (Table 1) were captured during a trapping effort of 11,034 trap/nights from 23 localities (9 cities, 6 villages and 8 natural areas) of the 29 sampled, covering 10 administrative regions in Chile and five bioclimatic regions (hyper-arid, arid, semi-arid, sub-humid and hyper-humid), latitude between −20.2167 and −53.1667 (Fig. 1). It was conducted from December 2015 to January 2018, during austral summer (December to February) and austral winter (July and September). These localities were selected based on the following demographic characteristics: (i) city, urban entity that has > 5000 inhabitants; (ii) village, urban entity with a population ranging between 2001–5000 inhabitants, or between 1001–2000 people, where less than 50% of the population that declares having worked, is engaged in primary activities (e.g. livestock, agriculture or fishing) [18]; and (iii) natural area, without human settlement, corresponding to national park (NP; unaltered areas of natural and biological diversity), and national reserves (NR; areas protecting wildlife populations or natural resources).

**Table 1:** Micromammal species captured, and fleas collected from 29 locations in Chile

Family and species of micromammal	No. micromammals collected	No. micromammals with fleas	No. of fleas collected	Prevalence (%)	Mean abundance	Mean intensity
				(95% CI)	(95% CI)	(95% CI)
Order Didelphimorphia						
Didelphidae						
*Thylamys elegans*	35	18	54	51.4 (33.98–68.62)	1.5 (0.83–2.97)	3.0 (1.83–5.22)
Order Rodentia						
Cricetidae						
*Abrothrix hirta*	319	191	643	59.9 (54.58–65.60)	2.0 (1.73–2.32)	3.4 (2.98–3.76)
*Abrothrix lanosus*	1	1	1	100	1.0	1.0
*Abrothrix longipilis*	5	4	9	80.0 (28.35–99.50)	1.8 (0.60–2.80)	2.3 (1.25–3.25)
*Abrothrix olivacea*	434	206	518	47.5 (42.68–52.29)	1.2 (1.03–1.37)	2.5 (2.27–2.80)
*Chelemys macronyx*	1	0	0	0	0	–
*Irenomys tarsalis*	1	0	0	0	0	–
*Loxodontomys micropus*	24	21	66	87.5 (67.63–97.35)	2.8 (1.96–3.79)	3.1 (2.38–4.24)
*Oligoryzomys longicaudatus*	229	81	162	35.4 (29.18–41.95)	0.7 (0.55–0.88)	2.0 (1.72–2.36)
*Phyllotis darwini*	120	49	133	40.8 (31.95–50.18)	1.1 (0.82–1.42)	2.7 (2.24–3.20)
*Phyllotis limatus*	2	0	0	0	0	–
*Reithrodon physodes*	5	2	6	40.0 (5.27–85.34)	1.2 (0.00–3.20)	3.0 (1.00–3.00)
Octodontidae						
*Octodon bridgesi*	1	1	2	100	2.0	2.0
*Octodon degus*	69	54	387	78.3 (66.69–87.30)	5.6 (4.20–7.78)	7.2 (5.56–9.93)
Abrocomidae						
*Abrocoma bennetti*	3	3	77	100	25.7 (5.00–45.00)	25.7 (5.00–45.00)
Muridae						
*Mus musculus*	11	2	0	18.2 (2.28–51.78)	0.2 (0.00–0.36)	1 (0.00–0.00)
*Rattus norvegicus*	2	0	0	0	0	–
*Rattus rattus*	250	73	214	29.2 (23.64–35.27)	0.9 (0.64–1.14)	2.9 (2.40–3.70)
Total	1512	706	2272	46.7 (44.12–49.20)	1.5 (1.38–1.66)	3.2 (2.99–3.59)

*Note:* The total number of rodents captured for each species, number of parasitized rodents, prevalence of fleas parasitizing rodents, total number of fleas collected, mean abundance, and mean intensity are indicated

*Abbreviation*: CI, confidence interval

Micromammals were captured using a Sherman trap (23 × 7.5 × 9 cm, Sherman Co., Tallahassee, USA) and wire-mesh traps (30 × 10 × 11 cm; Forma Ltd., Santiago, Chile) baited with oats. The associated use of both types of traps strongly reduced the likelihood of a species being present but not captured. Each locality was sampled for two consecutive nights. In each sampling locality, the traps were placed in four parallel lines approximately 100 m from each other, and each line was equipped with 50 traps set 10 m apart from each other. Only in cities, traps were used along lines with a 5–10 m inter-trap space, and the traps were placed outside the buildings. The rodents were removed from the traps according to standard techniques [19], and were subsequently anesthetized with ketamine:xilazine (1:1) [20]. Flea samples from rodents were collected by hand or with forceps from the host and placed into sterile cryovials tubes with 95% ethanol. For each rodent, the total number of extracted fleas was recorded (abundance); with these data, the overall mean infection intensity (the number of fleas collected from all species/number of infested hosts), the overall mean abundance of infection (the number of collected fleas from all species/total number of hosts), and prevalence (the proportion of infected hosts) were calculated. The micromammals were identified following Iriarte [21]. Micromammals were released after sampling, except for invasive rodents [black rat (*Rattus rattus*), Norway rat (*Rattus norvegicus*), and house mouse (*Mus musculus*)] that were euthanized by cervical dislocation [19].

### DNA extraction and PCR amplification

For DNA extraction, 5 fleas per host were selected, and when the number of fleas per host was less than 5, all the fleas were analyzed. Finally, DNA extraction was performed from 1315 fleas. Each flea was washed and cut between the third and fourth abdominal tergite with a scalpel. DNA was extracted from individual fleas using DNeasy Blood & Tissue Kit (Qiagen, Hilden, Germany) according to the manufacturer’s protocols. The incubation time was 5 h; following DNA extraction, the flea’s exoskeleton was recovered and stored in 96% ethanol to later mount and identify the flea species.

The presence of *Rickettsia* spp. was initially screened by polymerase chain reaction (PCR) using a short fragment of citrate synthase (*gltA*) gene (401 bp; Table 2) [22]. Thereafter, *gltA* positive samples were tested using three genes: *gltA* (830 bp) [22], *sca5* (*ompB*) [23], and we designed a set of primers for the β-subunit of RNA polymerase (*rpoB*) of *Rickettsia* sp. (GenBank: AF076436; Table 2). The amplification conditions were as follows: 5 min at 95 °C, 40 cycles of 30 s at 95 °C, 30 s of annealing temperature (see Table 2), 30 s at 72 °C, followed by a final extension of 5 min at 72 °C. The reactions were performed with GoTaq Green Master Mix 2X (Promega, Madison, USA) 12.5 µl, 5.5 µl of ultrapure nuclease-free water, 2 µl of forward primer (10 µM), 2 µl of reverse primer (10 µM), and 4 µl of DNA sample. The negative controls were carried out with ultrapure water, and the positive control was genomic DNA of *R. conorii* (AmpliRun® *Rickettsia conorii* DNA Control; Vircell, Granada, Spain). A selected number of *Rickettsia*-positive samples were purified and sequenced by the Macrogen Company (Seoul, Korea).

**Table 2:** Primer sequences and annealing temperatures used to detect *Rickettsia* spp.

Target gene	Primer name	Nucleotide sequence (5’–3’)	Annealing T (°C)	Product length (bp)
*ProgltA* (401)	CS-78_F	GCAAGTATCGGTGAGGATGTAAT	48^a^	401
	CS-323_R	GCTTCCTTAAAATTCAATAAATCAGGAT		
*gltA* (830)	CS-239_F	GCTCTTCTCATCCTATGGCTATTAT	48^a^	830
	CS-1069_R	CAGGGTCTTCGTGCATTTCTT		
*rpoB* (395)	RirpoB_F	CCGACTCATTACGGTCGCATTTGT	55.5	395
	RirpoB_R	CCCATCAAAGCACGGTTAGCATCA		
*sca5* (862)	120.M59F	CCGCAGGGTTGGTAACTGC	50^b^	862
	120.807R	CCTTTTAGATTACCGCCTAA		

^a^Labruna et al. [22]

^b^Roux & Raoult [23]

*Abbreviations*: F, forward; R, reverse; T, temperature

### Phylogenetic and BLAST analyses

All DNA sequences were edited and aligned using the Codon Code Aligner (CodonCode Corporation, Centerville, MA, USA). All sequences generated in this study were compared with those available on GenBank using the BLAST program (see http://www.ncbi.nlm.nih.gov/BLAST/). A Bayesian probabilities tree was created using MrBayes 3.2 based on *gltA* 830-bp gene fragment, using *Anaplasma phagocytophilum* as the outgroup. We used the GTR + G substitution model to reconstruct the tree and 10,000,000 bootstrap trials.

### Flea mounting and identification

After DNA extraction, each flea’s exoskeleton was recovered and mounted on glass slides using conventional procedures. The fleas were identified using a light microscope, taxonomic keys, and the descriptions of Johnson [24], and Sanchez & Lareschi [25]. Voucher specimens (slides) were catalogued in the Museo de Zoología at Universidad de Concepción (MZUC-UCCC, Concepción, Chile) under the accession numbers 46647–46667.

### Statistical analysis

The prevalence (percentage of micromammals parasitized with fleas) and abundance mean (mean number of fleas per host) in species of micromammals was calculated with total of samples of fleas collected (*n* = 2272), and confidence intervals (95% CI) were calculated, using bootstrap (2000 bootstrap replicates). The prevalence of *Rickettsia* (percentage of fleas infected with *Rickettsia*) was calculated based on the PCR results. We used generalized linear models (GLM) with binomial distribution and logit function to identify the variables that may explain *Rickettsia* prevalence in fleas. The explanatory variables analyzed were bioclimatic regions (hyper-arid; arid; semi-arid; sub-humid; and hyper-humid), location type (city; village and natural area) and season (summer and winter). First, we built a model that included all bioclimatic regions and then we built models for each bioclimatic region. To assess the relationship between the prevalence and sample size, a Spearman correlation analysis was performed. The Chi-square test or Fisher’s exact test (if an expected cell count was < 5) was used to evaluate the differences in the prevalence of *Rickettsia* among species of flea. A *P*-value < 0.05 was considered statistically significant. The data were analyzed using JMP software^®^ (SAS Institute Inc., Cary NC, USA).

### Nucleotide sequence accession numbers

*Rickettsia* sequences generated in this study were deposited in the NCBI GenBank database under the following accession numbers: MN630893-MN630962 (*gltA*); MN630963-MN630997 (*rpoB*) and MT834938-MT834942 (*sca5*).

## Results

A total of 2272 fleas were collected from 13 micromammal species, with an overall prevalence of 46.7% (*n* = 706). The overall mean abundance was 1.5 fleas per host and the overall mean intensity was 3.2 fleas per parasitized host (Table 1). Excluding the species in which < 20 individuals were sampled, the micromammals that presented the highest prevalence of fleas were *Loxodontomys micropus* (Austral greater mouse, 87.5%) and *Octodon degus* (Fence degu, 78.3%), and the lowest prevalence was found in *R. rattus* (29.2%). The abundance and mean intensity were higher in *O. degus* (Table 1). The marsupial *Thylamys elegans* (Llaca mouse-opossum) had a prevalence of fleas of 51.4%. All of the flea species found in *T. elegans* corresponded to species that were also found in rodents (Table 3).

**Table 3:** Flea species identified for each micromammal species collected in this study

Family/species of micromammal	Family of flea	Species of flea
Cricetidae		
*Abrothrix hirta*	Hystricopsyllidae	*Chiliopsylla allophyla* (Rothschild, 1915)
		*Ctenoparia inopinata* (Rothschild, 1909)
		*Ctenoparia topalIi* (Smit, 1963)
		*Ctenoparia jordani* (Smit, 1955)
	Ctenophthalmidae	*Neotyphloceras crassispina* (Rothschild, 1914)
		*Neotyphloceras pardinasi* (Sanchez & Lareschi, 2014)
		*Neotyphloceras* spp.
	Ceratophyllidae	*Nosopsyllus fasciatus* (Bosc d’Antic, 1800)
	Stephanocircidae	*Sphinctopsylla ares* (Rothschild, 1911)
	Rhopalopsyllidae	*Tetrapsyllus amplus* (Jordan & Rothschild, 1923)
		*Tetrapsyllus tantillus* (Jordan & Rothschild, 1923)
		*Tetrapsyllus rhombus* (Smit, 1955)
*Abrothrix lanosus*	Stephanocircidae	*Sphinctopsylla ares* (Rothschild, 1911)
*Abrothrix longipilis*	Ctenophthalmidae	*Neotyphloceras chilensis* (Lewis, 1976)
	Rhopalopsyllidae	*Tetrapsyllus corfidii* (Rothschild, 1904)
	Pulicidae	*Hectopsylla* spp.
*Abrothrix olivacea*	Hystricopsyllidae	*Ctenoparia inopinata* (Rothschild, 1909)
		*Ctenoparia jordani* (Smit, 1955)
		*Ctenoparia topalIi* (Smit, 1963)
	Ctenophthalmidae	*Neotyphloceras crassispina* (Rothschild, 1914)
		*Neotyphloceras chilensis* (Lewis, 1976)
		*Neotyphloceras pardinasi* (Sánchez & Lareschi, 2014)
		*Agastopsylla boxi* (Jordan & Rothschild, 1923)
	Ceratophyllidae	*Nosopsyllus fasciatus* (Bosc d’Antic, 1800)
	Stephanocircidae	*Sphinctopsylla ares* (Rothschild, 1911)
	Rhopalopsyllidae	*Ectinorus cocyti* (Rothschild, 1904)
		*Tetrapsyllus amplus* (Jordan & Rothschild, 1923)
		*Tetrapsyllus tantillus (*Jordan & Rothschild, 1923)
		*Tetrapsyllus rhombus* (Smit, 1955)
		*Tetrapsyllus corfidii* (Rothschild, 1904)
		*Listronius* spp.
	Pulicidae	*Hectopsylla* spp.
	Leptopsyllidae	*Leptopsylla segnis* (Schönherr, 1811)
*Oligoryzomys longicaudatus*	Hystricopsyllidae	*Ctenoparia inopinata* (Rothschild, 1909)
		*Ctenoparia topalIi* (Smit, 1963)
	Ctenophthalmidae	*Neotyphloceras chilensis* (Lewis, 1976)
		*Neotyphloceras crassispina* (Rothschild, 1914)
		*Neotyphloceras pardinasi* (Sánchez & Lareschi, 2014)
	Ceratophyllidae	*Nosopsyllus fasciatus* (Bosc d’Antic, 1800)
	Stephanocircidae	*Sphinctopsylla ares* (Rothschild, 1911)
	Rhopalopsyllidae	*Ectinorus chilensis* (Lewis, 1976)
		*Tetrapsyllus amplus* (Jordan & Rothschild, 1923)
		*Tetrapsyllus rhombus* (Smit, 1955)
	Leptopsyllidae	*Leptopsylla segnis* (Schönherr, 1811)
*Phyllotis darwini*	Ctenophthalmidae	*Neotyphloceras chilensis* (Lewis, 1976)
		*Neotyphloceras crassispina* (Rothschild, 1914)
	Stephanocircidae	*Sphinctopsylla ares* (Rothschild, 1911)
	Rhopalopsyllidae	*Delostichus* spp.
		*Delostichus phyllotis* (Johnson, 1957)
		*Delostichus smiti* (Jameson & Fulk, 1977)
		*Tetrapsyllus rhombus* (Smit, 1955)
		*Tetrapsyllus tantillus* (Jordan & Rothschild, 1923)
	Pulicidae	*Hectopsylla* spp.
	Tungidae	*Tunga* spp.
*Loxodontomys micropus*	Ctenophthalmidae	*Neotyphloceras* spp.
	Stephanocircidae	*Sphinctopsylla ares* (Rothschild, 1911)
Octodontinidae		
*Octodon bridgesi*	Rhopalopsyllidae	*Delostichus phyllotis* (Johnson, 1957)
		*Tetrapsyllus* spp.
*Octodon degus*	Ctenophthalmidae	*Neotyphloceras* spp.
		*Neotyphloceras chilensis* (Lewis, 1976)
	Rhopalopsyllidae	*Delostichus* spp.
		*Delostichus coxalis* (Rothschild, 1909)
		*Delostichus degus* (Beaucournu, Moreno & González, 2011)
		*Delostichus phyllotis* (Johnson, 1957)
		*Delostichus smiti* (Jameson & Fulk, 1977)
		*Ectinorus chilensis* (Lewis, 1976)
		*Tetrapsylllus corfidii* (Rothschild, 1904)
		*Tetrapsyllus tantillus* (Jordan & Rothschild, 1923)
Abrocomidae		
*Abrocoma bennetti*	Ctenophthalmidae	*Neotyphloceras* spp.
		*Neotyphloceras chilensis* (Lewis, 1976)
	Rhopalopsyllidae	*Delostichus* spp.
		*Delostichus coxalis* (Rothschild, 1909)
		*Delostichus phyllotis* (Johnson, 1957)
		*Delostichus smiti* (Jameson & Fulk, 1977)
		*Ectinorus chilensis* (Lewis, 1976)
		*Tetrapsyllus corfidii* (Rothschild, 1904)
Muridae		
*Rattus rattus*	Hystricopsyllidae	*Ctenoparia inopinata* (Rothschild, 1909)
		*Ctenoparia jordani* (Smit, 1955)
	Ctenophthalmidae	*Neotyphloceras* spp.
		*Neotyphloceras chilensis* (Lewis, 1976)
		*Neotyphloceras pardinasi* (Sánchez & Lareschi, 2014)
	Ceratophyllidae	*Nosopsyllus fasciatus* (Bosc d’Antic, 1800)
	Stephanocircidae	*Sphinctopsylla ares* (Rothschild, 1911)
		*Plocopsylla* spp.
		*Plocopsylla wolffsohni* (Rothschild, 1909)
	Rhopalopsyllidae	*Delostichus coxalis* (Rothschild, 1909)
		*Delostichus smiti* (Jameson & Fulk, 1977)
		*Tetrapsyllus rhombus* (Smit, 1955)
	Leptopsyllidae	*Leptopsylla segnis* (Schönherr, 1811)
	Pulicidae	*Xenopsylla cheopis* (Rothschild, 1903)
		*Hectopsylla* spp.
*Mus musculus*	Leptopsyllidae	*Leptopsylla segnis* (Schönherr, 1811)
Order Didelphimorphia		
Didelphidae		
*Thylamys elegans*	Stephanocircidae	*Sphinctopsylla ares* (Rothschild, 1911)
	Ctenophthalmidae	*Neotyphloceras* spp.
		*Neotyphloceras chilensis* (Lewis, 1976)
		*Neotyphloceras crassispina* (Rothschild, 1914)
	Rhopalopsyllidae	*Delostichus smiti* (Jameson & Fulk, 1977)
		*Tetrapsyllus tantillus* (Jordan & Rothschild, 1923)

Of all collected fleas, 1315 flea specimens were analyzed, corresponding to 27 species from 15 genera and 8 families (Table 4). The most abundant flea species were *Sphinctopsylla ares* (*n* = 211) and *Neotyphloceras chilensis* (*n* = 202; Table 4). The rodents that presented the greatest flea richness were *Abrothrix olivacea* (olive grass mouse, 17 spp.), *R. rattus* (14 spp.), *A. hirta* (long-haired grass mouse, 11 spp.), and *Oligoryzomys longicaudatus* (long-tailed pygmy rice rat, 11 spp.; Table 3). Natural areas were where the largest number of flea species (*n* = 25) and specimens were collected (*n* = 784), followed by villages (18 species, 349 specimens) and cities (18 species, 181 specimens). *Agastopsylla boxi*, *Ctenoparia jordani*, *C. topali*, *Ectinorus cocyti* and *Plocopsylla lewisi* were exclusive to natural areas (national parks and national reserves). Conversely, *Xenopsylla cheopis* was only found in one city (Iquique). *Neotyphloceras chilensis* and *S. ares* were the dominant species in natural areas (*N. chilensis* (*n* = 119); *S. ares* (*n* = 151)), and villages (*N. chilensis* (*n* = 83); *S. ares* (*n* = 50)), while *Nosopsyllus fasciatus* (*n* = 37), and *C. inopinata* (*n* = 25) were the most frequently collected in cities. *Leptopsylla segnis*, *N. fasciatus*, and *X. cheopis* are synanthropic rodent fleas [26], and were more abundant in cities than in villages and natural areas.

**Table 4:** *Rickettsia* prevalence detected on fleas for each gene used in the different flea species analyzed

Family and species of flea	No. of fleas analyzed	No. of fleas positive for gene fragment (Prevalence in %)			
		*gltA* 401 bp	*gltA* 830 bp	*rpoB* 395 bp	*sca5* 862 bp
Hystricopsyllidae					
*Chiliopsylla allophyla*	7	2 (28.6)	2 (28.6)	2 (28.6)	2 (28.6)
*Ctenoparia* spp.	20	0 (0.0)	0 (0.0)	0 (0.0)	0 (0.0)
*Ctenoparia inopinata*	85	1 (1.2)	1 (1.2)	1 (1.2)	1 (1.2)
*Ctenoparia topali*	2	0 (0.0)	0 (0.0)	0 (0.0)	0 (0.0)
*Ctenoparia jordani*	5	0 (0.0)	0 (0.0)	0 (0.0)	0 (0.0)
Ctenophthalmidae					
*Agastopsylla boxi*	3	0 (0.0)	0 (0.0)	0 (0.0)	0 (0.0)
*Neotyphloceras* spp.	128	40 (31.3)	7 (5.5)	10 (7.8)	0 (0.0)
*Neotyphloceras crassispina*	35	2 (5.7)	0 (0.0)	2 (5.7)	0 (0.0)
*Neotyphloceras chilensis*	202	71 (35.1)	29 (14.4)	29 (14.4)	0 (0.0)
*Neotyphloceras pardinasi*	43	7 (16.3)	3 (7.0)	5 (11.6)	0 (0.0)
Ceratophyllidae					
*Nosopsyllus fasciatus*	52	7 (13.5)	1 (1.9)	2 (3.8)	0 (0.0)
Stephanocircidae					
*Sphinctopsylla ares*	211	20 (9.5)	16 (7.6)	19 (9.0)	2 (0.9)
*Plocopsylla* spp.	4	2 (50.0)	0 (0.0)	0 (0.0)	0 (0.0)
*Plocopsylla wolffsohni*	2	0 (0.0)	0 (0.0)	0 (0.0)	0 (0.0)
*Plocopsylla lewisi*	1	0 (0.0)	0 (0.0)	0 (0.0)	0 (0.0)
Rhopalopsyllidae					
*Delostichus* spp.	12	0 (0.0)	0 (0.0)	0 (0.0)	0 (0.0)
*Delostichus degus*	22	0 (0.0)	0 (0.0)	0 (0.0)	0 (0.0)
*Delostichus coxalis*	53	0 (0.0)	0 (0.0)	0 (0.0)	0 (0.0)
*Delostichus phyllotis*	7	1 (14.3)	0 (0.0)	0 (0.0)	0 (0.0)
*Delostichus smiti*	85	0 (0.0)	0 (0.0)	0 (0.0)	0 (0.0)
*Ectinorus* spp.	1	0 (0.0)	0 (0.0)	0 (0.0)	0 (0.0)
*Ectinorus cocyti*	1	0 (0.0)	0 (0.0)	0 (0.0)	0 (0.0)
*Ectinorus chilensis*	12	1 (8.3)	0 (0.0)	0 (0.0)	0 (0.0)
*Tetrapsyllus* spp.	11	0 (0.0)	0 (0.0)	0 (0.0)	0 (0.0)
*Tetrapsyllus amplus*	17	0 (0.0)	0 (0.0)	0 (0.0)	0 (0.0)
*Tetrapsyllus tantillus*	93	10 (10.8)	1 (1.1)	1 (1.1)	0 (0.0)
*Tetrapsyllus corfidii*	16	0 (0.0)	0 (0.0)	0 (0.0)	0 (0.0)
*Tetrapsyllus rhombus*	74	8 (10.8)	6 (8.1)	7 (9.5)	1 (1.4)
*Listronius* spp.	3	0 (0.0)	0 (0.0)	0 (0.0)	0 (0.0)
Tungidae					
*Tunga* spp.	4	0 (0.0)	0 (0.0)	0 (0.0)	0 (0.0)
Pulicidae					
*Hectopsylla* spp.	30	1 (3.3)	0 (0.0)	0 (0.0)	0 (0.0)
*Xenopsylla cheopis*	11	0 (0.0)	0 (0.0)	0 (0.0)	0 (0.0)
Leptopsyllidae					
*Leptopsylla segnis*	63	1 (1.6)	0 (0.0)	0 (0.0)	0 (0.0)
Total	1315	174 (13.2)	66 (5.0)	78 (5.9)	6 (0.5)

### Rickettsiae prevalence on fleas

Fifteen flea species were found to be *Rickettsia*-positive for the short fragment (401 bp) of the *gltA* gene, 9 for the long fragment (830 bp) of the *gltA* gene, 10 for the *rpoB* gene, and 4 for the *sca5* gene (Table 4). The highest prevalence (13.2%) was detected with the *gltA* 401-bp gene, followed by the *rpoB* (5.9%), *gltA* 830-bp (5.0%) and *sca5* (0.5%) genes (Table 4). Among the flea species in which more than 20 individuals were analyzed, the prevalence varied between 0–35.1%. The *Neotyphloceras* spp. had the highest prevalence of *Rickettsia* (*gltA* 401-bp = 29.4%, *gltA* 830-bp = 9.56%, and *rpoB* = 11.25%; Table 4). The four fragments (*gltA* 401-bp, *gltA* 830-bp, *rpoB* and *sca5*) showed significant differences in the prevalence of detected *Rickettsia* (*χ*^2^ = 193.207, *df* = 3, *P* < 0.001), exception for *gltA* 830-bp and *rpoB*, which did not show significant differences (*χ*^2^ = 1.934, *df* = 1, *P* = 0.164). No association was found between the number of fleas analyzed and the prevalence of *Rickettsia* detected for any of the genes analyzed (*rpoB*: ρ = 0.4267, *P* = 0.12; *gltA*: ρ = 0.3757, *P* = 0.18; *sca5*: ρ = 0.3272, *P* = 0.35).

According to the GLM analysis, the prevalence of *Rickettsia* infection was significantly higher in the semi-arid region (27.8%). In addition, the overall prevalence was significantly higher in the winter (20.6%) than in the summer (5.3%). The prevalence of *Rickettsia* was higher in natural areas (15.9%), and cities exhibited a marginally significant lower prevalence (4.97%) compared to the other two location types (village: 11.2%; Table 5). Comparisons between bioclimatic regions showed that in the arid region, the prevalence of *Rickettsia* was higher in the natural areas and in the winter. While in the semi-arid region, the highest prevalence occurred in the winter (73.7%), and the highest prevalence of *Rickettsia* was detected in the natural areas (77.8%), differentiating from the cities (14.0%). In the sub-humid region, there was no effect of the factors on the prevalence of *Rickettsia*, whereas in the hyper-humid region, we detected *Rickettsia* (5.49%) only in the natural areas.

**Table 5:** Generalized linear models (GLM) of *Rickettsia* prevalence

Modell	Model performance			Model component				
	L-R *χ*^2^	*df*	Prob > *χ*^2^	Source of variation	Estimate	SE	L-R *χ*^2^	*P*-value
All bioclimatic regions	102.61	7	< 0.0001*	Intercept	2.51	0.33	60.16	< 0.0001*
				Season (winter)	− 0.83	0.12	49.71	< 0.0001*
				Bioclimatic region (arid)	− 0.29	0.34	0.76	0.3840
				Bioclimatic region (hyper-arid)	1.00	1.24	0.65	0.4205
				Bioclimatic region (hyper-humid)	− 0.18	0.46	0.14	0.7001
				Bioclimatic region (semi-arid)	− 1.40	0.37	14.07	0.0002*
				Location type (natural area)	− 0.42	0.15	8.02	0.0046*
				Location type (city)	0.45	0.23	3.57	0.0588^#^
Arid	62.80	3	< 0.0001*	Intercept	2.31	0.20	314.21	< 0.0001*
				Season (winter)	− 0.87	0.16	44.07	< 0.0001*
				Location type (natural area)	− 0.56	0.19	10.98	0.0009*
				Location type (city)	0.50	0.31	3.40	0.0652
Semi-arid	65.52	3	< 0.0001*	Intercept	0.81	0.42	3.89	0.0484*
				Season (winter)	− 2.27	0.455	56.73	< 0.0001*
				Location type (natural area)	0.62	0.48	1.73	0.1880
				Location type (city)	− 0.99	0.79	1.35	0.2445
Sub-humid	4.45	3	0.2167	Intercept	2.96	0.33	266.75	< 0.0001*
				Season (winter)	0.36	0.22	2.66	0.1026
				Location type (natural area)	− 0.37	0.36	1.65	0.1992
				Location type (city)	0.69	0.58	2.36	0.1241
Hyper-humid	5.08	2	0.0788	Intercept	3.51	0.69	128.38	< 0.0001*
				Location type (natural area)	− 1.08	0.73	5.09	0.0240*
				Location type (city)	0.38	1.09	0.00	1.0000

*Abbreviations*: L-R, likelihood ratio; *df*, degrees of freedom; SE, standard error; *P ≤ 0.05, ^#^marginally significant

### BLAST analysis and phylogenetic inference

A total of 167 sequences of *gltA* 401-bp (*n* = 68), *gltA* 830-bp (*n* = 40), *rpoB* (*n* = 54) and *sca5* (*n* = 5) genes were analyzed (Table 6). For *gltA* 401-bp, out of the 68 sequences, 28 isolated from *Delostichus phyllotis* (*n* = 1), *L. segnis* (*n* = 1), *N. crassispina* (*n* = 1), *N. pardinasi* (*n* = 3), *Neotyphloceras* spp. (*n* = 7), *N. fasciatus* (*n* = 3), *Plocopsylla* sp. (*n* = 2), *S. ares* (*n* = 3), *T. rhombus* (*n* = 1) and *Tetrapsyllus tantillus* (*n* = 6) were 100% identical to *Rickettsia* sp. (GenBank: KY705378) obtained from the tick *Amblyomma parvitarsum*. Another 19 *gltA* sequences (401-bp) detected in *Neotyphloceras* spp. (*n* = 16), *Chiliopsylla allophyla* (*n* = 2) and *C. inopinata* (*n* = 1) were closely related to *Rickettsia* sp. MEAM1 (99%; GenBank: CP016305) isolated from whitefly *Bemisia tabaci* (Hemiptera: Aleyrodidae) (*n* = 16) and *Rickettsia* sp. Gr15 (GenBank: KP675966) detected in the tick *Hyalomma marginatum* (*n* = 3). Twenty-one sequences amplified from *Neotyphloceras* spp. (*n* = 1), *S. ares* (*n* = 13) and *T. rhombus* (*n* = 6) showed 97–98% identity with *Rickettsia* sp. (GenBank: U59712) isolated from *Adelia bipunctata* (Coleoptera: Coccinellidae). One sequence amplified from *S. ares* showed 93% similarity with uncultured *Rickettsia* sp. (GenBank: KY433588) detected in a tick.

**Table 6:** Similarity percentage for obtained sequences with BLAST analyses

Flea collection				Identification by gene sequence (% similarity with the corresponding sequence on GenBank)							
				*gltA* 401 bp		*gltA* 830 bp		*rpoB* 395 bp		*sca5* 862 bp	
Flea species	Total sequences amplified per flea	Host	Collection site	*Rickettsia* spp.	No. positive	*Rickettsia* spp.	No. positive	*Rickettsia* spp.	No. positive	*Rickettsia* spp.	No. positive
*Chiliopsylla allophyla*	2	*Abrothrix hirta*	Nonguén NR	*Rickettsia* sp. Gr15 (99%; KP675966)	2	“*Candidatus* Rickettsia senegalensis” (99%; KU499847)	2	*Rickettsia* sp. (100%; JF966777)	2	*Rickettsia felis* (94%; GQ385243)	2
*Ctenoparia inopinata*	1	*Abrothrix hirta*	Los Queules NR	*Rickettsia* sp. Gr15 (97%; KP675966)	1	*Rickettsia* sp. (99%; KY799066)	1	*Rickettsia* sp. (97%; JF966777)	1	*Rickettsia felis* (94%; GQ385243)	1
*Delostichus phyllotis*	1	*Octodon degus*	La Campana NP	*Rickettsia* sp. (100%; KY705378)	1	na		na			
*Leptopsylla segnis*	1	*Rattus rattus*	Lolol	*Rickettsia* sp. (100%; KY705378)	1	na		na		na	
*Neotyphloceras chilensis*	4	*Phyllotis darwini*	Las Chinchillas NR	*Rickettsia* sp. MEAM1 (99%; CP016305)	4	*Rickettsia* sp. (97%; KF646706)	4	*Rickettsia* sp. MEAM1 (97–98%; CP016305)	4	na	
	2	*Abrothrix olivacea*	Las Chinchillas NR	*Rickettsia* sp. MEAM1 (98–99%; CP016305)	2	*Rickettsia* sp. (97%; KF646706)	2	*Rickettsia* sp. MEAM1 (98%; CP016305)	2	na	
	1	*Abrothrix olivacea*	Fray Jorge NP	*Rickettsia* sp. MEAM1 (99%; CP016305)	1	*Rickettsia* sp. (97%; U76908)	1	*Rickettsia* sp. MEAM1 (98%; CP016305)	1	na	
	3	*Abrothrix olivacea*	Canela Baja	*Rickettsia* sp. MEAM1 (99%; CP016305)	2	*Rickettsia* sp. (97%; U76908)	3	*Rickettsia* sp. MEAM1 (98%; CP016305)	3	na	
	1	*Octodon degus*	Canela Baja	*Rickettsia* sp. MEAM1 (99%; CP016305)	1	*Rickettsia* sp. (97%; U76908)	1	*Rickettsia* sp. MEAM1 (98%; CP016305)	1	na	
	2	*Phyllotis darwini*	Canela Baja	*Rickettsia* sp. MEAM1 (99%; CP016305)	2	*Rickettsia* sp. (97%; U76908)	2	*Rickettsia* sp. MEAM1 (98-100%; CP016305)	2	na	
	1	*Thylamys elegans*	Canela Baja	*Rickettsia* sp. MEAM1 (99%; CP016305)	1	*Rickettsia* sp. (97%; U76908)	1	*Rickettsia* sp. MEAM1 (98%; CP016305)	1	na	
*Neotyphloceras crassispina*	1	*Oligoryzomys longicaudatus*	Laguna Torca NR	*Rickettsia* sp. (100%; KY705378)	1	na		na		na	
	1	*Oligoryzomys longicaudatus*	Cobquecura	na		na		*Rickettsia* sp. (94%; KX300203)	1	na	
	1	*Abrothrix hirta*	Cobquecura	na		na		*Rickettsia* sp. (94%; KX300203)	1	na	
*Neotyphloceras pardinasi*	2	*Abrothrix hirta*	Cobquecura	na		na		*Rickettsia* sp. (94–96%; KX300157)	2	na	
	1	*Abrothrix olivacea*	Cobquecura	na		na		*Rickettsia* sp. (94%; KX300157)	1	na	
	1	*Rattus rattus*	La Campana NP	*Rickettsia* sp. (100%; KY705378)	1	na		na		na	
	1	*Rattus rattus*	Quirihue	na		na		*Rickettsia* sp. (94%; KX300203)	1	na	
	2	*Rattus rattus*	Laguna Torca NR	*Rickettsia* sp. (99–100%; KY705378)	2	na				na	
	1	*Rattus rattus*	Canela Baja	*Rickettsia* sp. (100%; KY705378)	1	*Rickettsia* sp. (97%; U76908)	1	*Rickettsia* sp. MEAM1 (93%; CP016305)	1	na	
*Neotyphloceras* spp.	1	*Oligoryzomys longicaudatus*	Cobquecura	na		na		*Rickettsia* sp. (95%; KX300157)	1	na	
	1	*Abrothrix hirta*	Laguna Torca NR	*Rickettsia* sp. (100%; KY705378)	1	na		na		na	
	3	*Abrothrix olivacea*	Laguna Torca NR	*Rickettsia* sp. (100%; KY705378)	3	na		na		na	
	1	*Oligoryzomys longicaudatus*	Laguna Torca NR	*Rickettsia* sp. (99%; KY705378)	1	na		na		na	
	2	*Thylamys elegans*	Laguna Torca NR	*Rickettsia* sp. (100%; KY705378)	2	na		na		na	
	2	*Abrothrix olivacea*	Canela Baja	*Rickettsia* sp. MEAM1 (99%; CP016305)	2	*Rickettsia* sp. (97%; U76908)	2	*Rickettsia* sp. MEAM1 (98%; CP016305)	2	na	
	1	*Abrothrix olivacea*	Fray Jorge NP	*Rickettsia* sp. MEAM1 (99%; CP016305)		Rickettsia sp. (97%; U76908)	1	na		na	
	1	*Abrothrix hirta*	Los Queules NR	*Rickettsia* sp. (97%; U59712)	1	na		*Rickettsia* sp. (91%; JF966777)	1	na	
	1	*Abrothrix olivacea*	Los Queules NR	na		na		*Rickettsia* sp. (94%; KX300157)	1	na	
	1	*Thylamys elegans*	Fray Jorge NP	na		*Rickettsia* sp. (97%; U76908)	1	na		na	
*Nosopsyllus fasciatus*	2	*Rattus rattus*	Lolol	*Rickettsia* sp. (100%; KY705378)	2	na		na		na	
	1	*Rattus rattus*	Santa Cruz	*Rickettsia* sp. (100%; KY705378)	1	na		na		na	
*Plocopsylla* spp.	1	*Rattus rattus*	La Campana NP	*Rickettsia* sp. (100%; KY705378)	1	na		na		na	
	1	*Rattus rattus*	Til Til	*Rickettsia* sp. (100%; KY705378)	1	na		na		na	
*Sphinctopsylla ares*	6	*Abrothrix hirta*	Los Queules NR	*Rickettsia* sp. (97%; U59712)	6	*Rickettsia* sp. (98%; AJ269522)	5	*Rickettsia* sp. (91%; JF966777)	5	na	
	2	*Abrothrix hirta*	Cobquecura	*Rickettsia* sp. (98%; U59712)	2	*Rickettsia* sp. (98%; AJ269522)	1	*Rickettsia* sp. (91%; JF966777)	2	na	
	1	*Abrothrix hirta*	Coyhaique NR	*Rickettsia* sp. (98%; U59712)	1	*Rickettsia* sp. (98%; AJ269522)	1	*Rickettsia* sp. (93%; JF966777)	1	na	
	3	*Abrothrix olivacea*	Coyhaique NR	*Rickettsia* sp. (97%; U59712)	3	*Rickettsia* sp. (98%; AJ269522)	3	*Rickettsia* sp. (93%; JF966777)	3	na	
	2	*Abrothrix olivacea*	Los Queules NR	*Rickettsia* sp. (98%; U59712)	1	*Rickettsia* sp. (96%; KY799066)	2	*Rickettsia* sp. (91–93%; JF966777)	3	*Rickettsia hoogstraalii* (88%; EF629536)	2
	2	*Abrothrix olivacea*	Cobquecura	na				*Rickettsia* sp. (95%; KX300157)	2	na	
	1	*Abrothrix olivacea*	Cobquecura	Rickettsia sp. (93%; KY433588)	1	na		*Rickettsia* sp. (95%; KX300157)	1	na	
	1	*Oligoryzomys longicaudatus*	Cobquecura	na		na		*Rickettsia* sp. (95%; KX300157)	1	na	
	2	*Rattus rattus*	Laguna Torca NR	*Rickettsia* sp. (100%; KY705378)	2	na		na		na	
	1	*Thylamys elegans*	Laguna Torca NR	*Rickettsia* sp. (100%; KY705378)	1	na		na		na	
*Tetrapsyllus rhombus*	1	*Abrothrix hirta*	Los Queules NR	*Rickettsia* sp. (98%; U59712)	1	*Rickettsia* sp. (98%; AJ269522)	1	*Rickettsia* sp. (91%; JF966777)	1	na	
	1	*Abrothrix hirta*	Cobquecura	*Rickettsia* sp. (97%; U59712)	1	*Rickettsia* sp. (98%; AJ269522)	1	*Rickettsia* sp. (91%; JF966777)	1	na	
	3	*Abrothrix olivacea*	Los Queules NR	*Rickettsia* sp. (97%; U59712)	3	*Rickettsia* sp. (98%; AJ269522)	3	*Rickettsia* sp. (91%; JF966777)	3	na	
	1	*Abrothrix olivacea*	Los Queules NR	na		na		*Rickettsia* sp. (94%; KX300203)	1	na	
	1	*Abrothrix olivacea*	Coyhaique NR	*Rickettsia* sp. (97%; U59712)	1	*Rickettsia* sp. (98%; AJ269522)	1	*Rickettsia* sp. (93%; JF966777)	1	na	
	1	*Rattus rattus*	La Campana NP	*Rickettsia* sp. (100%; KY705378)	1	na		na		na	
*Tetrapsyllus tantillus*	4	*Abrothrix olivacea*	Lolol	*Rickettsia* sp. (100%; KY705378)	4	na		na		na	
	1	*Abrothrix olivacea*	Til Til	*Rickettsia* sp. (100%; KY705378)	1	na		na		na	
	1	*Octodon degus*	La Campana NP	*Rickettsia* sp. (100%; KY705378)	1	na		na		na	

*Note:* The table shows genes, GenBank accession numbers, similarity percent (%), flea species positive for *Rickettsia*, micromammal flea hosts, and locations where fleas were collected

*Abbreviations*: NR, National Reserve; NP, National Park; na, no amplification

Two sequences of *gltA* 830-bp segments showed high identity (99%) to “*Candidatus* Rickettsia senegalensis” (GenBank: KU499847) previously identified in a cat flea (*C. felis*). Forty sequences obtained from *S. ares* (*n* = 12), *T. rhombus* (*n* = 6), *Neotyphloceras* spp. (*n* = 19) and *C. inopinata* (*n* = 1) shared 97–98% identity with *Rickettsia* spp. (GenBank: KF646706; KY799066; U76908; and AJ269522) isolated from the insects *Nesidiocoris tenuis* (Heteroptera: Miridae), *Mansonia uniformis* (Diptera: Culicidae), *Empoasca papayae* (Hemiptera: Cicadellidae) and *Adalia decempunctata* (Coleoptera: Coccinellidae).

Seventeen amplified *rpoB* sequences in *Neotyphloceras* spp. shared 93–100% similarity with *Rickettsia* sp. MEAM1 (GenBank: CP016305) isolated from *B. tabaci*. Another 24 sequences derived from *C. allophyla* (*n* = 2), *C. inopinata* (*n* = 1), *Neotyphloceras* spp. (*n* = 1), *S. ares* (*n* = 14) and *T. rhombus* (*n* = 6) showed between 91% and 100% homology with *Rickettsia* sp. (GenBank: JF966777) of *Synosternus pallidus* (Siphonaptera: Pulicidae). Nine amplified sequences from *Neotyphloceras* spp. (*n* = 9) were 94–96% similar to *Rickettsia* sp. (GenBank: KX300157) isolated from a bat (*Myotis emarginatus*). Finally, 4 sequences isolated from *Neotyphloceras* spp. (*n* = 3) and *T. rhombus* (*n* = 1) showed lower homology with *Rickettsia* sp. (94%, GenBank: KX300203) isolated from a bat (*Eptesicus serotinus*).

Three *sca5* fragments isolated from *C. allophyla* (*n* = 2) and *C. inopinata* (*n* = 1) showed homology with *R. felis* (94%; GenBank: GQ385243), and 2 fragments detected from *S. ares* showed low identity to *R. hoogstraalii* (GenBank: EF629536) (Table 6).

**Fig. 2:**
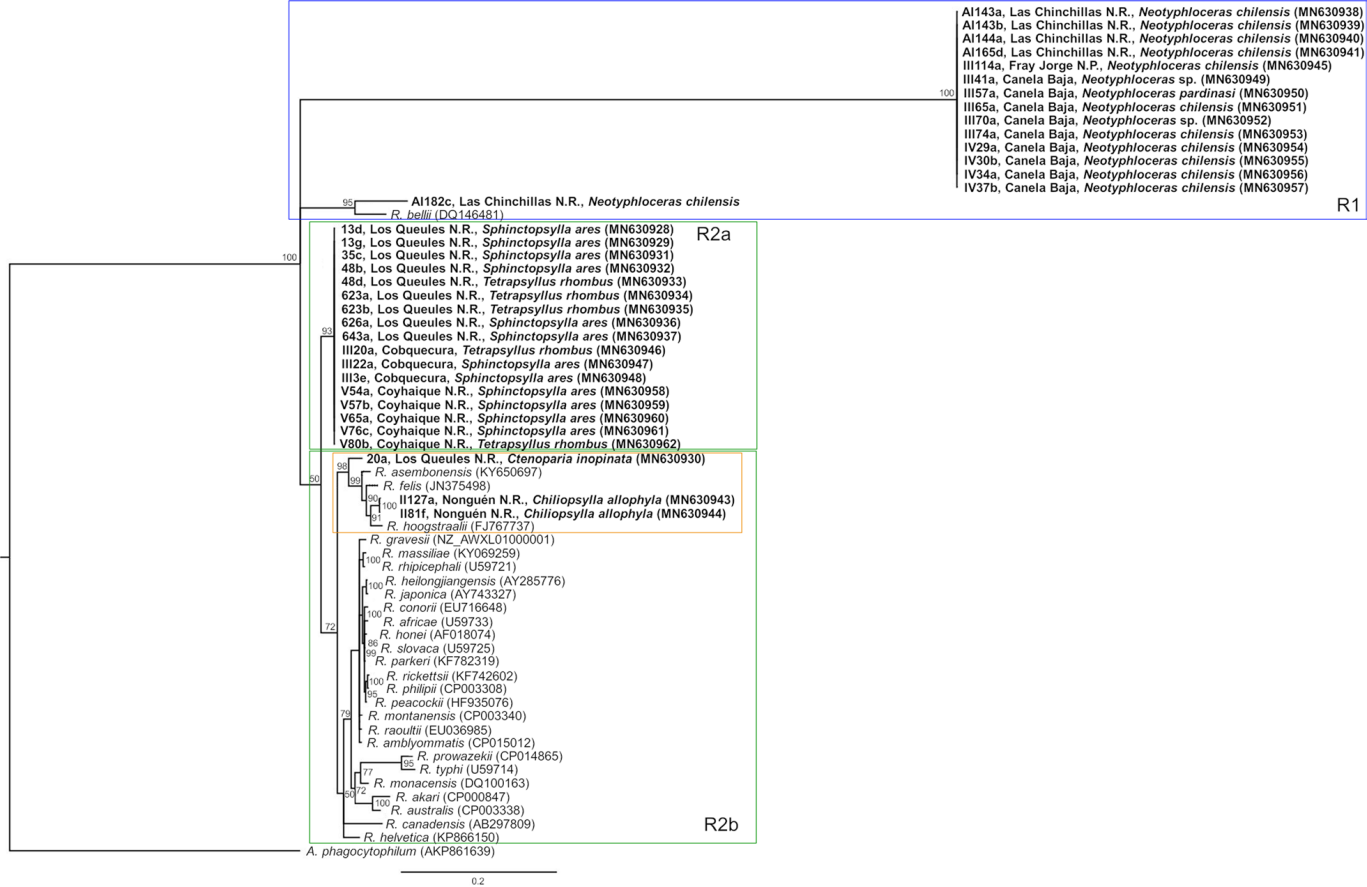
Phylogenetic tree of *gltA* 830-bp gene of *Rickettsia*. The values on each node show the Bayesian probability of each clade. The accession number for each sequence is indicated. Flea species and locality are indicated for the sequences generated in this study. The principal clades are labelled R1, R2a and R2b

The phylogenetic tree shows two well-differentiated clades with 100% nodal support (Fig. 2). Clade R1 was formed by sequences obtained from *Neotyphloceras* fleas collected in Las Chinchillas NR (31°30′36″S, 71°05′15″W), Canela Baja (31°23′54″S, 71°27′27″W), and Fray Jorge NP (30°23′S, 71°23′W). *Rickettsia bellii* (GenBank: DQ146481) was positioned on a basal branch in this group. The clade R2 was subdivided into two subclades: R2a and R2b. R2a, with 93% nodal support, is related to sequences obtained from *T. rhombus* and *S. ares* collected in Los Queules NR, Cobquecura, and Coyhaique NR, comprising a larger area of distribution (latitude: − 35° to − 45°S) than clade R1. Subclade R2b was formed by sequences obtained from *C. inopinata* and *C. allophyla* collected in Los Queules NR and Nonguén NR, respectively. The newly generated sequences were positioned closely to *R. hoogstraalii* (GenBank: FJ767737) isolated from *Haemaphysalis sulcata* (tick) in Croatia [27], *R. asembonensis* detected in *C. felis* from Peru (GenBank: KY650697) [28] and *R. felis* isolated from *C. felis* in Brazil (GenBank: JN375498) [29].

## Discussion

To the best of our knowledge, we have provided for the first time evidence for the presence of *Rickettsia* DNA in 15 flea species identified on wild micromammals and synanthropic rodents in Chile. The prevalence of *Rickettsia* spp. infections in fleas varied between species of flea, bioclimatic regions, seasons and location type. We found a higher prevalence in winter, the semi-arid region and natural areas.

The fleas were characterized as being highly host-opportunistic, occupying various host species [7]. This is confirmed by our study, since of the 27 flea species collected, 19 parasitized more than one species of micromammal. We also highlight the high flea species richness recorded in *R. rattus*, where 10 of the 14 species identified in this rodent correspond to the flea species identified on native rodents. This rodent was mainly captured in urban areas; however, we also found it in rural and natural areas, this occurs mainly because these rodents have an omnivore diet and plasticity in their behavior, characteristics that allow them to inhabit a great diversity of environments, adapting successfully to urban, rural and wild environments [30, 31]. *Rickettsia-*positive fleas parasitizing *R. rattus* in these three areas indicate that this species could play a key role in spreading the disease from wild to urban environments [16, 32]. Conversely, we also observed that wild species enter human-occupied environments since they provide shelter and food. *Abrothrix olivacea* was the most frequently captured wild species in urban and rural areas and had the highest flea richness and the highest number of *Rickettsia*-positive fleas. This species has been described to have a “random walk” type of dispersal behavior, so it can easily go from wild to domestic environments [33]. These findings are important because these rodent species could act as “bridge hosts” and aid in the spread of the disease [32, 34]. On the other hand, in natural areas, the rodent species most frequently captured was *A. hirta*; this species, like *A. olivacea*, had a high prevalence of *Rickettsia*-positive fleas. This rodent decreased its presence in areas with human intervention, which is consistent with the findings reported by Monteverde & Hadora [33], who described that this rodent preferably moves within the wild environment. Rodent populations can act as “source populations” and may be involved in the direct transmission of the pathogen to the target population [34].

The prevalence of *Rickettsia* spp. infections detected in our study was variable (0–35%), and associated with the identity of the flea species, season, type of locality and bioclimatic area. However, similar differences have been reported in other studies. For example, Radzijevskaja et al. [35] reported different prevalence related to the flea species analyzed (range: 0–43%). Also, Kuo et al. [36] carried out an extensive sampling analyzing the presence of *Rickettsia* in six species of flea, reporting 0–12.1% of prevalence in the different species of flea analyzed. Furthermore, flea infestations in this study were generally higher during the winter; however, this did not occur in all bioclimatic areas. Other studies have found similar results, attributing this variation to the differences in the seasonal reproductive cycles of the different species of flea [37], which are unknown in most of the species found in this study. On the other hand, the higher prevalence of *Rickettsia* in fleas detected in natural areas can be explained by the greater diversity of species of micromammals and, therefore, of fleas. Thus, the differences in the prevalence of infection in the different species of flea, localities, seasons and bioclimatic zones found in our study, reveal the importance of the composition of the community, both fleas, and hosts, in determining the prevalence of *Rickettsia* in fleas, and therefore in the risk of infection in areas with different human disturbance.

In this study, we found two well-differentiated clades with a high degree of support. Clade R1 is formed by sequences obtained from fleas of the genus *Neotyphloceras*, collected from rodents *Phyllotis darwini*, *A. olivacea*, *O. degus*, *R. rattus*, and the marsupial *T. elegans* from central-north Chile (latitude: − 30° to − 31°S). This clade is related to *R. bellii* and is described as an ancestral group of *Rickettsia* [38], and which exhibits some specificity concerning its host [39]. This supports our results, where only bacteria detected in *Neotyphloceras* were found in this clade. *Rickettsia bellii* is endosymbiont of hard (Ixodidae) and soft (Argasidae) ticks throughout the American continent [39]. It has been classified as non-pathogenic for animals and humans [40], although seropositive samples have been found in dog blood in Brazil; however, the pathogenic effect is unknown [41]. Experimentally, this bacterium grows easily in mammalian cells. In experimental inoculations in guinea pig and rabbit, it produces, depending on the inoculated dose received, from a mild inflammatory reaction to necrotic scabs a typical symptomatology of other pathogenic rickettsiae [29]. Furthermore, it is capable of producing antibodies in experimental infections in the big-eared opossum *Didelphis aurita*, but without rickettsemia [42]. These results indicate that some flea species present in wild and synanthropic micromammals could carry a new ancestral genotype of *Rickettsia*, just like those reported by Song et al. [43] in China from fleas of wild rodents.

The R2 clade was divided into two large groups, R2a and R2b. R2a grouped all of the sequences detected in fleas being extracted from two species of flea, *S. ares* (Stephanocircidae) and *T. rhombus* (Rhopalopsyllidae), which were obtained from villages and natural environments through wide latitudinal distribution (latitude of − 35° to − 45°S). This corresponds to the wide distribution of the hosts of infected fleas (*A. hirta* and *A. olivacea*). Conversely, R2b was formed by sequences obtained from *C. allophyla* and *C. inopinata* belonging to the same family (Hystricopsylidae); both species of flea were collected in wild rodents (*A. hirta* and *A. olivacea*) from wild areas (Los Queules NR and Nonguén NR) in the south-central zone of Chile. These sequences are closely related to *R. hoogstraalii*, *R. asembonensis* and *R. felis*, all of which are members of the spotted fever group rickettsiae (SFG) [28, 29, 38]. The SFG consists of > 30 species that can be found worldwide, most of them with pathogenic effects on humans [44]. Our analysis showed a close relationship with *R. hoogstraalii*, a widely distributed bacterium that is still unknown for its pathogenicity in humans. This bacterium has been detected in both hard ticks (*H. punctata*, *H. sulcate* and *H. parva*) and soft ticks (*Ornithodoros moubata*, *Carios capensis*, *C. sawaii* and *Argas persicus*) present in domestic animals, bird nests, vegetation, and human dwellings [3, 45–47]. A similar situation occurs with *R. asembonensis*. It also has a wide distribution worldwide, having been reported in North America and South America, Asia, the Middle East and Europe [48], although it is associated with a greater number of ectoparasites, including fleas, ticks, and mites of domestic and peridomestic animals (*C. canis*, *C. felis*, *X. cheopis*, *Pulex irritans*, *Amblyomma ovale*, *Rhipicephalus sanguineus*, *R. microplus* and *Ornithonysus bacoti*) [49–53]. It has also been detected in monkey blood in Malaysia [54] and in dog blood in South Africa [55]. Although these bacteria live in parasitic arthropods close to humans and are closely associated with *R. felis*, there is no evidence yet of possible infection or pathogenicity [48]. On the other hand, *R. felis* is an emergent, widely distributed, flea-borne human pathogen, and like *R. asembonensis* and *R. hoogstraalii*, is associated with domestic and peridomestic animals and their ectoparasites [56, 57]. The main vector is *C. felis*, although mosquitoes (*Anopheles gambiae*) have also been detected as competent vectors [58]. Unlike *R. asembonensis* and *R. hoogstraalii*, this bacterium is of known pathogenicity causing fever, fatigue, nausea, muscle aches, back pain, headaches, macular rash, joint pain and eschar [49]. Although the BLAST analysis showed a low percentage of similarity with *R. felis* (*sca5* 94%), the phylogenetic analysis shows a close relationship with *Rickettsia* detected in *C. allophyla* in south-central Chile. Until now, in Chile, only *R. felis* has been registered in *C. felis* [12].

## Conclusions

To the best of our knowledge, our study reports, for the first time in Chile, the presence of *Rickettsia* in different species of parasitic fleas of wild micromammals and invasive rodents found in both natural and human environments. Moreover, there is evidence of at least two clades of *Rickettsia* associated with fleas. These data increase the knowledge of possible *Rickettsia* vectors/reservoirs in Chile. However, greater efforts should be made to monitor and determine the degree of pathogenicity of the detected rickettsiae.

## Data Availability

Not applicable.

## Abbreviations

MTG: major typhus group
MSFG: major spotted fever group
TG: typhus group
AG: ancestral group
NP: national park
NR: national reserve
GLM: generalized linear models
SAG: Servicio Agricola y Ganadero
CONAF: Corporación Nacional Forestal
PCR: polymerase chain reaction
BLAST: Basic Local Alignment Search Tool
MZUC-UCCC: Museo de Zoología Universidad de Concepción
CI: confidence interval
*n*: number of individuals
*df*: degrees of freedom

## References

[1] Walker DH, Quah SR (2016). Rickettsia. International encyclopedia of public health.

[2] Schumacher L, Snellgrove A, Levin ML (2016). Effect of *Rickettsia rickettsii* (Rickettsiales: Rickettsiaceae) infection on the biological parameters and survival of its tick vector—*Dermacentor variabilis* (Acari: Ixodidae). J Med Entomol..

[3] Blanton LS, Walker DH (2017). Flea-borne rickettsioses and rickettsiae. Am J Trop Med Hyg..

[4] Shpynov SN, Fournier PE, Pozdnichenko NN, Gumenuk AS, Skiba AA (2018). New approaches in the systematics of rickettsiae. New Microbes New Infect..

[5] Perlman SJ, Hunter MS, Zchori-Fein E (2006). The emerging diversity of *Rickettsia*. Proc R Soc..

[6] Weinert LA, Werren JH, Aebi A, Stone GN, Jiggins FM (2009). Evolution and diversity of *Rickettsia* bacteria. BMC Biol..

[7] Bitam I, Dittmar K, Parola P, Whiting MF, Raoult D (2010). Fleas and flea-borne diseases. Int J Infect Dis..

[8] Feng AYT, Himsworth CG (2014). The secret life of the city rat: a review of the ecology of urban Norway and black rats (*Rattus norvegicus* and *Rattus rattus*). Urban Ecosyst..

[9] Himsworth CG, Parsons KL, Jardine C, Patrick DM (2013). Rats, cities, people, and pathogens: a systematic review and narrative synthesis of literature regarding the ecology of rat-associated zoonoses in urban centers. Vector Borne Zoonotic Dis..

[10] Beaucournu JC, Moreno L, González-Acuña D (2016). Le genre *Dasypsyllus* Baker 1905: description d’une espèce nouvelle et essai de mise au point sur ce genre (Siphonaptera: Ceratophyllidae). Ann la Soc Entomol Fr..

[11] Beaucournu JC, Moreno L, González-Acuña D (2014). Fleas (Insecta-Siphonaptera) of Chile: a review. Zootaxa..

[12] Labruna MB, Ogrzewalska M, Moraes-Filho J, Lepe P, Gallegos JL, López J (2007). *Rickettsia felis* in Chile. Emerg Infect Dis..

[13] Poo-Muñoz DA, Elizondo Patrone MC, Escobar LE, Astorga F, Bermúdez S, Martinez-Valdebenito CP (2016). Fleas and ticks in carnivores from a domestic-wildlife interface: implications for public health and wildlife. J Med Entomol..

[14] Müller A, Rodríguez E, Walker R, Bittencourt P, Pérez-Macchi S, Ricardo Gonçalves L (2013). Occurrence and genetic diversity of *Bartonella* spp. (Rhizobiales: Bartonellaceae) and *Rickettsia* spp. (Rickettsiales: Rickettsiaceae) in cat fleas (Siphonaptera: Pulicidae) from Chile. J Med Entomol..

[15] Cevidanes A, Di Cataldo S, Vera F, Lillo P, Millán J (2018). Molecular detection of vector-borne pathogens in rural dogs and associated *Ctenocephalides felis* fleas (Siphonaptera: Pulicidae) in Easter Island (Chile). J Med Entomol..

[16] Moreno L, Espinoza-Carniglia M, Lizama-Schmeisser N, Torres LG, Silva-De La Fuente MC, Lareschi M (2019). Fleas of black rats (*Rattus rattus*) as reservoir host of *Bartonella* spp. in Chile. PeerJ..

[17] Meerburg BG, Singleton GR, Kijlstra A (2009). Rodent-borne diseases and their risks for public health rodent-borne diseases and their risks for public health. Crit Rev Microbiol..

[18] Instituto Nacional de Estadisticas (Chile). Ciudades, pueblos, aldeas y caseríos. 2005. http://arks.princeton.edu/ark:/88435/dsp019g54xm55n. Accessed 22 Apr 2015.

[19] Mills JN, Yates TL, Childs JE, Parmenter RR, Ksiazek TG, Rollin PE (1995). Guidelines for working with rodents potentially infected with Hantavirus. J Mammal..

[20] Carpenter J, Marion C (2018). Exotic animal formulary.

[21] Iriarte A (2008). Los mamíferos de Chile.

[22] Labruna MB, Whitworth T, Horta MC, Bouyer DH, McBride JW, Pinter A (2004). *Rickettsia* species infecting *Amblyomma cooperi* ticks from an area in the state of São Paulo, Brazil, where brazilian spotted fever is endemic. J Clin Microbiol..

[23] Roux V, Raoult D (2000). Phylogenetic analysis of members of the genus *Rickettsia* using the gene encoding the outer-membrane protein r*OmpB* (*ompB*). Int J Syst Evol Microbiol..

[24] Johnson P (1957). A classification of the Siphonaptera of South America, with descriptions of new species.

[25] Sánchez J, Lareschi M (2014). Two new species of *Neotyphloceras* (Siphonaptera: Ctenophthalmidae) from Argentinean Patagonia. Zootaxa..

[26] Lewis RE (1998). Résumé of the Siphonaptera (Insecta) of the World. J Med Entomol..

[27] Duh D, Punda-Polic V, Avsic-Zupanc T, Bouyer D, Walker DH, Popov VL (2010). *Rickettsia hoogstraalii* sp. nov., isolated from hard-and soft-bodied ticks. Int J Syst Evol Microbiol..

[28] Loyola S, Flores-Mendoza C, Torre A, Kocher C, Melendrez M, Luce-Fedrow A (2018). *Rickettsia asembonensis* characterization by multilocus sequence typing of complete genes, Peru. Emerg Infect Dis..

[29] Ogata H, La Scola B, Audic S, Renesto P, Blanc G, Robert C (2006). Genome sequence of *Rickettsia bellii* illuminates the role of amoebae in gene exchanges between intracellular pathogens. PLoS Genet..

[30] Medina-Pinto RA, Torres-Castro MA, Medina-Pinto RA, Bolio-González ME, Rodríguez-Vivas RI (2019). Natural cysticercus fasciolaris infection in rodents from a rural area in Yucatan, Mexico. Vet Mex..

[31] Whisson DA, Quinn JH, Collins KC (2007). Home range and movements of roof rats (*Rattus rattus*) in an old-growth riparian forest, California. J Mammal..

[32] Ahmed S, Dávila JD, Allen A, Tacoli And C, Fèvre EM (2019). Does urbanization make emergence of zoonosis more likely? Evidence, myths and gaps. Environ Urban..

[33] Monteverde Hadora (2017). Movimientos de roedores intra- e inter-ambiente y riesgo de exposición al Hantavirus “Andes” en Patagonia norte, Argentina. Ecol Austral..

[34] Caron A, Cappelle J, Cumming GS, de Garine-Wichatitsky M, Gaidet N (2015). Bridge hosts, a missing link for disease ecology in multi-host systems. Vet Res..

[35] Radzijevskaja J, Kaminskiene E, Lipatova I, Mardosaite-Busaitiene D, Balčiauskas L, Stanko M (2018). Prevalence and diversity of *Rickettsia* species in ectoparasites collected from small rodents in Lithuania. Parasit Vectors..

[36] Kuo CC, Huang JL, Lin TE, Wang HC (2012). Detection of *Rickettsia* spp and host and habitat associations of fleas (Siphonaptera) in eastern Taiwan. Med Vet Entomol..

[37] Kim HC, Yang YC, Chong ST, Ko SJ, Lee SE, Klein TA (2010). Detection of *Rickettsia typhi* and seasonal prevalence of fleas collected from small mammals in the republic of Korea. J Wildl Dis..

[38] Merhej V, Raoult D (2011). Rickettsial evolution in the light of comparative genomics. Biol Rev..

[39] Krawczak FS, Labruna MB, Hecht JA, Paddock CD, Karpathy SE (2018). Genotypic characterization of *Rickettsia bellii* reveals distinct lineages in the United States and South America. Biomed Res Int..

[40] Parola P, Paddock CD, Socolovschi C, Labruna MB, Mediannikov O, Kernif T (2013). Update on tick-borne rickettsioses around the world: a geographic approach. Clin Microbiol Rev..

[41] Fortes FS, Silveira I, Moraes-Filho J, Leite RV, Bonacim JE, Biondo AW (2010). Seroprevalence of *Rickettsia bellii* and *Rickettsia felis* in dogs, São José dos Pinhais, State of Paraná, Brazil. Rev Bras Parasitol Vet..

[42] Horta MC, Sabatini GS, Moraes-Filho J, Ogrzewalska M, Canal RB, Pacheco RC (2010). Experimental infection of the opossum *Didelphis aurita* by *Rickettsia felis*, *Rickettsia bellii*, and *Rickettsia parkeri* and evaluation of the transmission of the infection to ticks *Amblyomma cajennense* and *Amblyomma dubitatum*. Vector Borne Zoonotic Dis..

[43] Song S, Chen C, Yang M, Zhao S, Wang B, Hornok S (2018). Diversity of *Rickettsia* species in border regions of northwestern China. Parasit Vectors..

[44] Robinson MT, Satjanadumrong J, Hughes T, Stenos J, Blacksell SD (2019). Diagnosis of spotted fever group *Rickettsia* infections: the Asian perspective. Epidemiol Infect..

[45] Karasartova D, Gureser AS, Gokce T, Celebi B, Yapar D, Keskin A (2018). Bacterial and protozoal pathogens found in ticks collected from humans in Corum province of Turkey. PLoS Negl Trop Dis..

[46] Brinkmann A, Hekimoǧlu O, Dinçer E, Hagedorn P, Nitsche A, Ergünay K (2019). A cross-sectional screening by next-generation sequencing reveals *Rickettsia*, *Coxiella*, *Francisella*, *Borrelia*, *Babesia*, *Theileria* and *Hemolivia* species in ticks from Anatolia. Parasit Vectors..

[47] Chisu V, Leulmi H, Masala G, Piredda M, Foxi C, Parola P (2017). Detection of *Rickettsia hoogstraalii*, *Rickettsia helvetica*, *Rickettsia massiliae*, *Rickettsia slovaca* and *Rickettsia aeschlimannii* in ticks from Sardinia. Italy. Ticks Tick Borne Dis..

[48] Maina AN, Jiang J, Luce-Fedrow A, St. John HK, Farris CM, Richards AL (2019). Worldwide presence and features of flea-borne *Rickettsia asembonensis*. Front Vet Sci..

[49] Jiang J, Maina AN, Knobel DL, Cleaveland S, Laudisoit A, Wamburu K (2013). Molecular detection of *Rickettsia felis* and *Candidatus* Rickettsia asembonensis in fleas from human habitats, Asembo, Kenya. Vector Borne Zoonotic Dis..

[50] Luce-Fedrow A, Maina AN, Otiang E, Ade F, Omulo S, Ogola E (2015). Isolation of *Candidatus* Rickettsia asembonensis from *Ctenocephalides* fleas. Vector Borne Zoonotic Dis..

[51] Kocher C, Morrison AC, Leguia M, Loyola S, Castillo RM, Galvez HA (2016). Rickettsial disease in the Peruvian Amazon basin. PLoS Negl Trop Dis..

[52] Troyo A, Moreira-Soto RD, Calderon-Arguedas Ó, Mata-Somarribas C, Ortiz-Tello J, Barbieri ARM (2016). Detection of rickettsiae in fleas and ticks from areas of Costa Rica with history of spotted fever group rickettsioses. Ticks Tick Borne Dis..

[53] Dall’Agnol B, Souza U, Webster A, Weck B, Stenzel B, Labruna M (2017). “*Candidatus* Rickettsia asembonensis” in *Rhipicephalus sanguineus* ticks. Brazil. Acta Trop..

[54] Tay ST, Koh FX, Kho KL, Sitam FT (2015). Rickettsial infections in monkeys. Malaysia. Emerg Infect Dis..

[55] Kolo AO, Sibeko-Matjila KP, Maina AN, Richards AL, Knobel DL, Matjila PT (2016). Molecular detection of zoonotic Rickettsiae and *Anaplasma* spp. in domestic dogs and their ectoparasites in Bushbuckridge, South Africa. Vector Borne Zoonotic Dis..

[56] Stevenson HL, Labruna MB, Montenieri JA, Kosoy MY, Gage KL, Walker DH (2005). Detection of *Rickettsia felis* in a New World flea species, *Anomiopsyllus nudata* (Siphonaptera: Ctenophthalmidae). J Med Entomol..

[57] Panti-May JA, Torres-Castro M, Hernández-Betancourt S, Dzul-Rosado K, Zavala-Castro J, López-Avila K (2015). Detection of *Rickettsia felis* in wild mammals from three municipalities in Yucatan, Mexico. Ecohealth..

[58] Dieme C, Bechah Y, Socolovschi C, Audoly G, Berenger JM, Faye O (2015). Transmission potential of *Rickettsia felis* infection by *Anopheles gambiae* mosquitoes. Proc Natl Acad Sci USA.

